# Prucalopride reduces the number of reflux episodes and improves subjective symptoms in gastroesophageal reflux disease: a case series

**DOI:** 10.1186/1752-1947-8-34

**Published:** 2014-02-05

**Authors:** Simon Nennstiel, Monther Bajbouj, Roland M Schmid, Valentin Becker

**Affiliations:** 1 Klinikum rechts der Isar der Technischen Universität München, II. Medizinische Klinik und Poliklinik, Ismaninger Strasse 22, 81675 München, Germany

**Keywords:** GERD, Gastroesophageal reflux, Non-acid reflux, PPI-persistent reflux, Prucalopride, pH or multichannel impedance (MII) monitoring

## Abstract

**Introduction:**

Treatment of persistence to proton pump inhibitors or non-acid reflux episodes in patients with gastroesophageal reflux disease is challenging. Prucalopride, a selective high affinity serotonin (5-HT_4_) receptor agonist, might offer a possible new therapeutic alterative.

**Case presentations:**

We report four chronically constipated female gastroesophageal reflux disease-patients with reflux symptoms and an increased number of reflux episodes in combined esophageal pH and multichannel impedance monitoring treated with prucalopride (2mg per day). Symptoms were persistent to proton pump inhibitors and ranitidine. Gastroesophageal reflux was detected by pH or multichannel impedance (MII) monitoring. Numbers of all reflux episodes as well as non-acid reflux episodes were reduced in all of our patients. The objective findings were concordant with subjective reports of symptom relief. There were no major adverse events in any patient during therapy with prucalopride.

**Conclusion:**

Administration of prucalopride showed promising results in the treatment of persisting or weakly and/or non-acid reflux episodes in our case series in four constipated patients. Therefore, prucalopride can be regarded as a possible therapeutic option in the treatment of standard proton pump inhibitor-persistent reflux in the chronically constipated patient. However, further prospective trials are needed to prove our findings.

## Introduction

Gastroesophageal reflux disease (GERD) is a relevant disorder in western countries with increasing frequency and incidence [1]. Most patients with acid reflux episodes do adequately respond to proton pump inhibitor (PPI) therapy [2]. More challenging is treatment of “PPI-persistent” or weakly and/or non-acid reflux episodes, which may also cause relevant reflux symptoms [3]. In persistent symptoms to standard PPI therapy, high-dose PPI therapy or baclofen, a GABA B agonist, are reported to reduce weakly and/or non-acid gastroesophageal reflux episodes in single cases [4]. Whereas double doses of PPIs are an appropriate therapy with significant reduction of liquid and/or mixed reflux events [5], available data related to baclofen therapy are inconclusive and symptom responder rates are poor [6].

In the past, cisapride, a procinetic non-selective serotonin (5-HT_4_) receptor agonist, was used with good amelioration of reflux symptoms [7]. Therapeutic effects might be explained by a decrease in transient lower esophageal sphincter relaxation, enhanced lower esophageal motor activity, and enhanced gastric or duodenal emptying [8-11]. However, due to severe cardiac side-effects (QT-time prolongation), cisapride was removed from the market in the year 2000 [12]. Prucalopride, a new selective high affinity serotonin (5-HT_4_) receptor agonist, was introduced on the German market in 2010. It is approved in the therapy of chronic constipation in women and stimulates colonic movement [13]. In addition, possible effects on upper intestinal motility similar to cisapride are conceivable. So far, no reports on the effect of prucalopride in GERD patients are available.

Combined esophageal pH or multichannel impedance (MII) monitoring [14] allows detection of all types of reflux events [15], which enables the characterization of persistent gastroesophageal reflux episodes [16]. This method has been shown to be highly sensitive [14]. Therefore, pH and/or MII is a suitable technique to objectively evaluate pathological reflux episodes in patients with non-acid reflux episodes.

GERD is defined as esophageal acid exposure >5% in 24 hour pH monitoring [17] or the number of total reflux episodes greater than 73 in MII monitoring [18].

In the presented case report, we provide experiences with four patients with PPI-persistent or weakly and/or non-acid reflux episodes treated with prucalopride due to chronic constipation.

Four female Caucasian patients from our outpatient department with chronic constipation and typical reflux symptoms (heartburn), despite standard PPI therapy for at least four weeks, were asked to perform pH and/or MII monitoring before initiation of and during prucalopride therapy. Our patients completed a symptom-based questionnaire before pH and/or MII monitoring in each case. This standardized questionnaire asked the patients about typical and atypical GERD-symptoms, the frequency of these symptoms and the subjective evaluation of the influence of these symptoms on daily life. This previously published questionnaire [5] focuses on subjective parameters instead of another objective scoring system, such as the Frequency Scale for the Symptoms of GERD (FSSG) [19]. To evaluate objective parameters, combined MII and pH monitoring was performed using an ambulatory, multi-channel, intra-luminal impedance system, consisting of a portable data logger and a combined pH impedance catheter (Tecnomatix ZAN S 61C 01 E, Sandhill Scientific, Highlands Ranch, CO, USA). Six impedance electrodes as well as a distal pH-antimony probe were placed at pre-defined spots on this catheter (3.0cm, 5.0cm, 7.0cm, 9.0cm, 15.0cm and 17.0cm; pH probe 5.0cm). The catheter was placed with the pH antimon probe located 5cm above the manometrically-defined lower esophagus sphincter. Data were recorded for 21 hours and 23 hours, respectively.

Gastroesophageal reflux was detected by impedance changes was defined on the basis of previous reports [1,2,18]. Reflux episodes were defined as either acidic or non-acidic, and if a retrograde bolus movement was detected by impedance and pH value was below or above 4, respectively. Furthermore, the content of the reflux episode was characterized according to its composition (gas, fluid or mixed). Meals were excluded from analysis.

## Case presentations

The characteristics of each patient are summarized in Table 1. Results of pH or MII monitoring before and after prucalopride treatment and associated subjective symptom scores are displayed in Tables 2 and 3.

**Table 1:** Patients’ characteristics

	**Patient 1**	**Patient 2**	**Patient 3**	**Patient 4**
Age (years)	49	50	70	40
BMI (Du Bois)	35.9kg/m^2^	26.8kg/m^2^	21.9kg/m^2^	33.8kg/m^2^
GERD symptoms	Heartburn, regurgitation	Heartburn, regurgitation	Regurgitation	Heartburn, globus, bloating
GERD history	- GERD symptoms >10 years - symptoms persistent to standard PPI	- GERD symptoms >20 years - symptoms persistent to PPI	- GERD symptoms >10 years - symptoms persistent to standard PPI	- GERD symptoms for three months
	- subjective impression of improved symptom control to treatment with ranitidine			- GERD symptoms persistent after successful *H. pylori* eradication and ongoing standard PPI treatment
				- start prucalopride six months after eradication
Endoscopic findings	Small axial herniation, no reflux lesions	Small axial herniation, ERD LA C	Small axial herniation, no reflux lesions	No axial herniation, no reflux lesions, *H. pylori* gastritis

BMI – body mass index, ERD – erosive reflux disease, GERD – gastroesophageal reflux disease, H.p. – Helicobacter pylori, LA – Los Angeles classification, PPI – proton pump inhibitor.

**Table 2:** Results of multichannel impedance-pH monitoring before pucalopride treatment

	**Patient 1**		**Patient 2**		**Patient 3**		**Patient 4**	
MII-pH monitoring before treatment with prucalopride	Medication during monitoring:		Medication during monitoring:		Medication during monitoring:		Medication during monitoring:	
	- Ranitidine 75mg per day		- Pantoprazole 40mg per day		- Omeprazole 20mg per day		- none	
	Acid reflux time:	1.5%	Acid reflux time:	0.3%	Acid reflux time:	0.1%	Acid reflux time:	2.8%
	Overall reflux episodes:	128	Overall reflux episodes:	143	Overall reflux episodes:	96	Overall reflux episodes:	108
	Mixed or liquid reflux episodes:	94	Mixed or liquid reflux episodes:	140	Mixed or liquid reflux episodes:	79	Mixed or liquid reflux episodes:	104
	Gaseous reflux episodes:	34	Gaseous reflux episodes:	3	Gaseous reflux episodes:	17	Gaseous reflux episodes:	4
	Acid reflux episodes:	84	Acid reflux episodes:	15	Acid reflux episodes:	17	Acid reflux episodes:	71
	Non- and/or weakly acid reflux episodes:	44	Non- and/or weakly acid reflux episodes:	128	Non- and/or weakly acid reflux episodes:	79	Non- and/or weakly acid reflux episodes:	37
*Scale 0 to 10: 0 = none; 10 = unbearable	Subjective symptom scores*		Subjective symptom scores*		Subjective symptom scores*		Subjective symptom scores*	
	- Heartburn + Regurgitation: 5		- Heartburn + Regurgitation: 7		- Regurgitation: 5		- Heartburn, Globus, Bloating: 9	
	- Limitation of daily life: 5		- Limitation of daily life: 6		- Limitation of daily life: 5		- Limitation of daily life: 9	
	SI: positive for heartburn and regurgitation		SI: positive for heartburn and regurgitation		SI: positive for regurgitation		SI: positive for heartburn, globus and bloating	

MII – multichannel impedance monitoring, SI - symptom index.

Subjective symptom score is based on the questionnaire previously described by Becker V *et al.*[7]. SI, Symptom index.

**Table 3:** **Results of multichannel impedance-pH monitoring during pucalopride-treatment**[7]

	**Patient 1**		**Patient 2**		**Patient 3**		**Patient 4**	
MII and/or pH monitoring during treatment with prucalopride	Medication during monitoring:		Medication during monitoring:		Medication during monitoring:		Medication during monitoring:	
	- Pantoprazole 40mg per day		- Pantoprazole 40mg per day		- Omeprazole 20mg per day		- Prucalopride 2mg per day	
	+ Prucalopride 2mg per day		+ Prucalopride 2mg per day		+ Prucalopride 2mg per day			
	Acid reflux time:	0.1%	Acid reflux time:	0.9%	Acid reflux time:	1.2%	Acid reflux time:	1.9%
	Overall reflux episodes:	46	Overall reflux episodes:	108	Overall reflux episodes:	83	Overall reflux episodes:	59
	Mixed or liquid reflux episodes:	5	Mixed or liquid reflux episodes:	100	Mixed or liquid reflux episodes:	74	Mixed or liquid reflux episodes:	40
	Gaseous reflux episodes:	41	Gaseous reflux episodes:	8	Gaseous reflux episodes:	9	Gaseous reflux episodes:	19
	Acid reflux episodes:	17	Acid reflux episodes:	32	Acid reflux episodes:	26		39
	Non- and/or weakly acid reflux episodes:	29	Non- and/or weakly acid reflux episodes:	76	Non- and/or weakly acid reflux episodes:	57	Acid reflux episodes: Non- and/or weakly acid reflux episodes:	20
*Scale 0 to10: 0 = none; 10 = unbearable	Subjective symptom scores*		Subjective symptom scores*		Subjective symptom scores*		Subjective symptom scores*	
	- Heartburn + Regurgitation: 3		- Heartburn + Regurgitation: 5		- Regurgitation: 3		- Heartburn, Globus, Bloating: 4	
	Limitation of daily life: 3		Limitation of daily life: 5		Limitation of daily life: 3		Limitation of daily life: 9	
	SI: positive for heartburn and regurgitation		SI: positive for heartburn, negative for regurgitation		SI: negative for regurgitation		SI: negative for heartburn, globus and bloating	

MII – multichannel impedance monitoring, SI – symptom index.

Subjective symptom scores are based on the questionnaire previously described by Becker V *et al.*[7]. SAP, Symptom association probability; SI, Symptom index.

There were no major adverse events in any patient during therapy with prucalopride.

### Patient 1

This patient, a 49-year-old Caucasian woman with chronic constipation and regular use of laxatives, reported having typical GERD-symptoms (heartburn, regurgitation) for over 10 years, persistent to standard PPI therapy. However, she had the impression of symptom improvement to therapy with ranitidine 75mg per day. Gastroscopy showed small axial herniation with signs of erosive reflux lesions. Radiologic fluoroscopy was performed due to intermittent dysphagia, but the test results showed no dysfunction of esophageal motility. First pH-monitoring on ranitidine-therapy showed normal findings; however, MII monitoring revealed elevated overall acid and non-acid reflux episodes (n = 128) with a positive symptom index (=reported symptoms in >50% associated to reflux-episodes; symptom index (SI)) for heartburn and regurgitation. Subjective severity of the symptoms on a 10-point scale was stated as “5”, and influence of the symptoms on daily life was stated as “5” by our patient. After this first measurement, standard PPI therapy was initiated; however, symptoms persisted. Thus, additional prucalopride medication was initiated.

The second pH or MII-monitoring after the initiation of prucalopride 2mg per day (plus pantoprazole 40mg per day) showed an overall decrease (n = 46), acid (from 84 to 17) and non- and/or weakly acid (from 44 to 29) reflux episodes. Her SI was still positive for heartburn and regurgitation; however, her subjective symptom score for these symptoms and the subjective score for limitation on her daily life both decreased to “3”.

### Patient 2

This 50-year-old Caucasian woman reported having chronic constipation and typical reflux symptoms (heartburn and regurgitation) for over 20 years. Daily PPI medication with pantoprazole 40mg per day did not result in relief of the symptoms. Gastroscopy revealed a small axial herniation, with erosive reflux disease (Los Angeles classification grade C). Due to reported dysphagia of solid and liquid food, a manometry was performed to exclude dysfunction of esophageal motility. The first pH monitoring showed normal findings, the MII monitoring showed an increase of overall reflux episodes (n = 143) with mainly non- and/or weakly acid reflux (n = 128). The symptom index was positive for heartburn and regurgitation, the subjective symptom score on a 10-point scale for these symptoms was “7” and the subjective score for the influence of these symptoms on daily life was “6”.

Combined pH and MII monitoring with additive prucalopride medication (2mg per day) showed a decrease of overall (n = 108) and non- and/or weakly acid reflux episodes (n = 76) and a slight increase of acid reflux episodes (from 15 to 32). Her SI was negative for regurgitation but remained positive for heartburn. Her subjective symptom score for heartburn and regurgitation and her subjective score for the influence of these symptoms on her daily life were both stated as a “5” by our patient.

### Patient 3

This 70-year-old Caucasian woman with chronic constipation reported having reflux symptoms (regurgitation) for more than 10 years. Medication with omeprazole 20mg per day did not result in any symptom relief. Gastroscopy showed small axial herniation without erosive lesions to the distal esophagus. During the first combined pH and MII-monitoring, her SI was positive for regurgitation and whereas pH monitoring showed normal findings, MII monitoring revealed elevated overall reflux episodes (n = 96) with foremost non- and/or weakly acid reflux episodes (n = 79). Her subjective symptom score for regurgitation was “5”. The subjective rating of the influence of regurgitation on her daily life was also “5” on a 10-point scale.

Prucalopride therapy led to a decrease of overall reflux episodes (n = 83), non- and/or weakly reflux episodes (n = 57). There was a slight increase of acid reflux episodes (from 17 to 26). Her subjective scores for symptoms and influence on daily life were both stated as “3”. SI for regurgitation was negative.

### Patient 4

This 40-year-old Caucasian woman with chronic constipation reported having heartburn, globus and bloating for three months. First gastroscopy showed no axial herniation or erosive reflux lesions; however, a *Helicobacter pylori-*induced gastritis was detected. Nevertheless, after successful eradication of *H. pylori* (confirmed by a C-13 breath test) and continued standard PPI therapy, symptoms persisted. Therefore, she quit PPI therapy and the first combined pH and MII monitoring was conducted without any medication. In pH monitoring we found non-pathologic values. MII monitoring revealed elevated overall reflux episodes (n = 108) and, in particular, elevated acid reflux episodes (n = 71). Her SI was positive for heartburn, globus and bloating. Her subjective assessment for these symptoms was “9” and the influence of these symptoms on her daily life was also stated as “9” by our patient.

The second MII monitoring, after initiation of prucalopride therapy, showed a decrease of symptoms overall (n = 59), acid (from 71 to 39) and non- and/or weakly acid (from 37 to 20) reflux episodes. Her SI was negative for heartburn, globus and bloating. The subjective score for these symptoms decreased to a “5”; however, subjective assessment of the influence of these symptoms on her daily life remained at “9”.

## Discussion

This case series indicates for the first time that prucalopride, single therapy or in addition to PPI medication, might relevantly reduce the number of “PPI-persistent” reflux episodes in patients with typical reflux symptoms and an increased number of reflux episodes, assessed by combined impedance and pH monitoring. The objective findings were concordant with subjective reports of symptom relief.

In our case series, numbers of all reflux episodes as well as non-acid reflux episodes were reduced in all of our patients. In acid reflux episodes, results are controversial. However, there were only a few episodes of acid reflux during the first measurement, so that the increase of these episodes can possibly be explained by physiologic reflux variability during the different measurements. Our other two patients (with initially pathologic elevated acid reflux episodes) showed a relevant reduction for this kind of reflux during prucalopride medication. Taking all objective and subjective parameters in the four patients together, there was good response to prucalopride therapy.

Prucalopride mainly effects colonic motility and, therefore, is used in the therapy of chronic constipation. Prior experiments in animals indicated that prucalopride also effects contractility of the stomach [20,21] Moreover, a positive effect on gastric motility was already observed in the chronic constipated patient. In our patients, prucalopride led to an accelerated overall gastric emptying and small bowel transit which we believe to be the main effect of prucalopride on GERD [22]. However, this effect could not be affirmed in healthy, non-constipated subjects [23]. In addition, we hypothesize an influence of prucalopride on upper gastrointestinal motility in analogue to the effect described in cisapride, including enhanced lower esophageal motor activity, a decrease of transient lower esophageal sphincter relaxation and enhanced gastric and/or duodenal emptying [8-11], at least in the constipated patient. In addition, a stimulation of esophageal body contraction and an elevation of lower esophagus sphincter (LES) resting pressure was also shown for mosapride, another selective 5-HT_4_-agonist [24], and is, therefore, used for GERD patients (in combination with PPI) in some Asian countries [25]. Of course, due to the different structures of these 5HT_4_ agonists, the characteristic effects of these drugs are not directly comparable, especially since prucalopride is a high affinity 5HT_4_ agonist mainly effecting colonic movement. However, there is evidence that prucalopride can have an effect on upper gastrointestinal motility (in the constipated patients) [22], even if this effect possibly is weaker than the effect of cisapride or mosapride. The effect of prucalopride on esophageal motility will need further objective evaluation, with high resolution (HR) manometry testing before and during prucalopride therapy. In fact, HR manometry would have been an excellent diagnostic tool in our case series and should be included in further trials.

As a main limitation to our study, the small number of patients in a single center setting needs to be mentioned. However, all of our patients were analyzed in a standard outpatient setting. Considerable subjective improvement of reflux symptoms after one week was reported in all of our patients. Still, we report a preliminary case series. The primary goal in this case series was to report the effect of prucalopride in GERD patients. For a final conclusion, double-blinded trials are needed.

## Conclusion

In conclusion, our case series showed a reduction of “PPI-persistent” and non-acid reflux episodes during prucalopride therapy in four chronically constipated women. Therefore, prucalopride might be a new and promising therapeutic option in the challenging demand for treating constipated patients with GERD who had standard PPI-persistent reflux episodes. Further prospective, placebo-controlled studies involving a larger number of patients and esophageal motility studies are needed to prove these findings and to evaluate if prucalopride could also be an appropriate therapy in the non-constipated GERD patient.

## Consent

Written informed consent was obtained from our patients for publication of this case series. A copy of the written consents is available for review by the Editor-in-Chief of this journal.

## Abbreviations

FSSG: Frequency scale for the symptoms of GERD [19]; GERD: Gastroesophageal reflux disease; HR manometry: High-resolution manometry; LES: Lower esophagus sphincter; MII: Multichannel impedance (monitoring); PPI: Proton pump inhibitor; QT-time: Time between the start of the Q wave and the end of the T wave in the electrocardiogram; SI: Symptom index (reported symptoms in >50% associated to reflux episodes).

## Competing interest

The authors declare that they have no conflict of interest.

## Authors’ contributions

SN collected the patients’ data, created contact or kept in contact with the patients, conducted pH and MII monitoring, participated in the design of the study and drafted the manuscript. MB participated in the design of the study and helped to draft the manuscript. RMS participated in the study’s coordination and helped in the development of the manuscript. VB conceived of the study and its design, participated in its coordination, conducted pH and MII monitoring and took part in the development of the manuscript. All authors read and approved the final manuscript.
